# An non-loglinear enzyme-driven law of photosynthetic scaling in two representative crop seedlings under different water conditions

**DOI:** 10.1038/s41598-020-69702-8

**Published:** 2020-07-29

**Authors:** Zhiwei Wang, Lingchao He, Kang Xu, Hanjian Hu, Alamgir Khan, Maozi Lin, Yan Li, Shun Liu, Genxuan Wang

**Affiliations:** 1 College of Life Sciences, Zhejiang University, Hangzhou, 310058 Zhejiang Province China; 2 Fuqing Branch of Fujian Normal University, Fuqing, 350300 Fujian Province China; 3 State Key Laboratory of Subtropical Silviculture, Zhejiang A&F University, Hangzhou, 311300 Zhejiang Province China

**Keywords:** Ecology, Plant sciences

## Abstract

The loglinear pattern of respiratory scaling has been studied for over a century, while an increasing number of non-loglinear patterns have been found in the plant kingdom. Several previous studies had attempted to reconcile conflicting patterns from the aspects of statistical approaches and developmental stages of the organisms. However, the underlying enzymatic mechanism was largely ignored. Here, we propose an enzyme-driven law of photosynthetic scaling and test it in typical crop seedlings under different water conditions. The results showed that the key enzyme activity, the relative photosynthetic assimilation and the relative growth rate were all constrained by the available water, and the relationship between these biological traits and the available water supported our predictions. The enzyme-driven law appears to be more suitable to explain the curvature of photosynthetic scaling than the well-established power law, since it provides insight into the biochemical origin of photosynthetic assimilation.

## Introduction

Photosynthesis is the fundamental metabolism for most of world’s ecosystems^1^. The scaling model of the power law has been widely employed to describe net primary production scaling^2–5^ in log–log space. Some researchers have applied log-transformed power law to dark respiration rates in trees^6–8^ and shrubs^9^. A few authors have used photosynthetic rates as metabolic indicators in ocean and plants^10,11^, but the subjects are marine systems and saplings, respectively. However, the relationship between photosynthetic rate and biomass in crop plants has not been studied under the light of power law.

Photosynthetic assimilation and respiration are both important metabolic processes, and they reverse each other. However, metabolic scaling of basic respiration is a longstanding problem^12,13^, and towards its resolution, a series of arguments about the power law and the ‘true’ value of scaling exponents have been suggested^14–17^. It has theoretically been shown that the scaling exponents should range between 0.31 and 1.00 in aquatic invertebrates and algae^18^. The models based on polynomial equation have been used to describe and quantify the double-log linear deviation in basic metabolic scaling^19,20^. However, there are still contentious in the origin of photosynthetic assimilation and the methods used to estimate parameters^21^.

Many log–log relationships of metabolic scaling have been shown to be nonlinear and cannot be represented by a simple power function^20–22^. Transformations of logarithms are referred to as patterns of “non-loglinear allometry”^23^ or patterns of “nonlinear allometry”^24^, which can be represented by adding other terms based on power laws^25–27^ or energy dynamics^13,28^. Models can explain the variation in scaling exponents in terms of resource allocation (Dynamic Energy Budget Model)^29^, metabolic level^30^ and thermodynamics processes^31^. A sigmoidal model predicted by the plant adaptive strategies hypothesis in NPP scaling^1^. However, although photosynthesis and respiration are a series of biochemical reactions, chemical kinetics is rarely used to explain the challenges of metabolic scaling. We have developed an enzyme-driven law (EDL), which was successfully applied to quantify the curvature and exponent variations of respiration scaling in microbes^32^. For terrestrial plants, photosynthesis differs significantly from respiration in microbes. Thus, a modified law is required to describe the photosynthetic scaling by incorporating biochemical mechanisms.

In this study, a new enzyme-driven law for photosynthetic scaling was derived from the hypothesis and both the relative rates of photosynthetic assimilation and growth were constrained by the key enzymatic activities. The law was tested by the data of photosynthesis and growth under different water conditions in represented crop seedlings (rice and maize^33,34^) to explain the biochemical origin of curvature in photosynthetic scaling.

## Results

### Key enzymatic constraint in relative photosynthetic assimilation and growth

**Figure 1:**
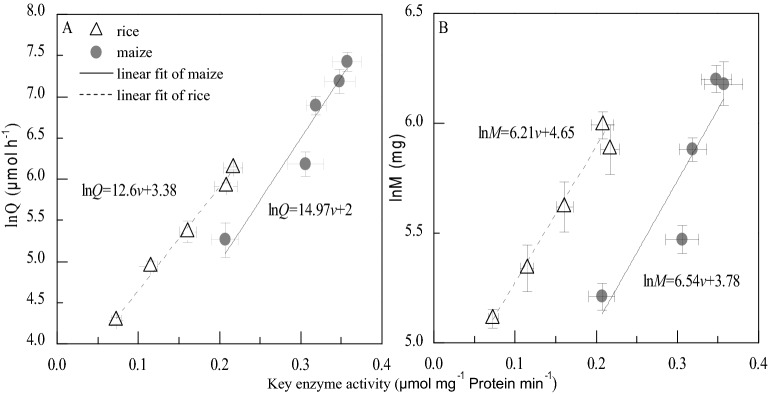
Both the relative metabolic rates (ln*Q*) (**A**) and relative change of growth (ln*M*) (**B**) vary with key enzyme activity linearly under experimental conditions. The total sample number of each treatment is fifteen.

Our experiment supported the basic hypothesis, both relative photosynthetic assimilation and growth were constrained by the activity of key enzymes (Eq. 1). The key enzyme activity (RuBPcase in rice, PEPcase in maize) increased linearly with relative photosynthetic assimilation (Fig. 1A), and relative change in body mass (Fig. 1B). The photosynthetic key enzyme drove the assimilation and growth respectively in both rice (C_3_) and maize (C_4_) with different parameters (Fig. 1).

### The water potential dependence of relative photosynthetic assimilation and relative growth

**Figure 2:**
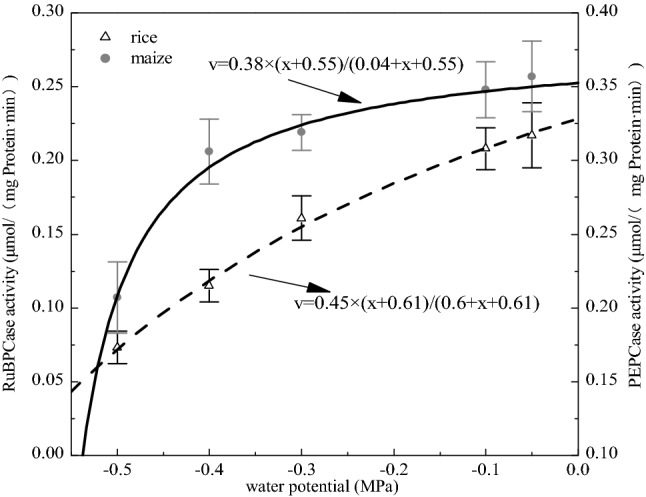
The activity of the two key enzymes changed with a gradient of available water nonlinearly. PEPCase activity in maize (solid circle) correspond to right vertical axis, and RuBPCase activity in rice (open triangle) correspond to left vertical axis. The total sample number of each treatment is fifteen.

The available water was the limited substrate constrained by the key enzyme in C_3_ (rice) and C_4_ (maize) plants under the experiment conditions. Key enzyme activities varied with available water in accordance with the Michaelis–Menten equation (Fig. 2), which meant that available water could be treated as a limiting substrate for the key pathway of photosynthesis at least for seeding stage. The K value of available water in RuBPcase (0.6) was bigger than that in PEPCase (0.04), which meant PEPCase was more sensitive to available water; the water potential of rice was lower than that in maize when the key enzyme activity was zero (Fig. 2).

As an important part of plants, water is directly involved in important metabolic processes in plants, one of the raw materials for plant photosynthesis and a medium for many important biochemical reactions. Meanwhile, water stress is one of the main reasons for crop yield reduction under drought conditions. Water potential could be steadily controlled relatively and affected photosynthesis and growth synchronously.

**Figure 3:**
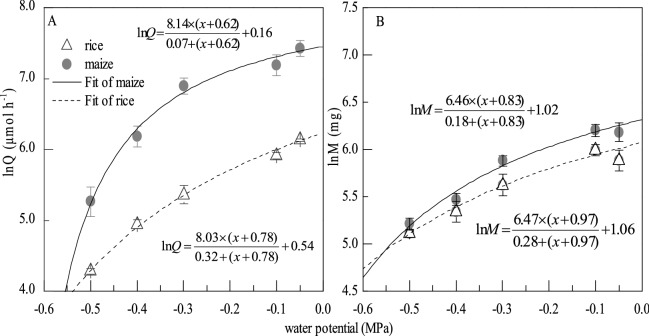
The water potential dependence of relative photosynthetic assimilation (**A**) and relative growth (**B**) under the experimental conditions. The total sample number of each treatment is fifteen.

The pattern of water potential dependence appeared in relative photosynthetic assimilation and relative growth at the individual level (Fig. 3). The trends of relative photosynthetic assimilation and relative growth versus water potential were consistent in both rice and maize. The *K*_*q*_ and *K*_*m*_ in rice (0.32, 0.28) were bigger than that in maize (0.07, 0.18), which meant maize was more sensitive to available water; The water potential in rice was lower than that in maize when the key enzyme activity was zero (Fig. 3). Equations 2 and 3 were checked and the results were in accordance with our predictions.

### The interdependence between relative photosynthetic assimilation (ln*Q)* and growth (ln*M*)

**Figure 4:**
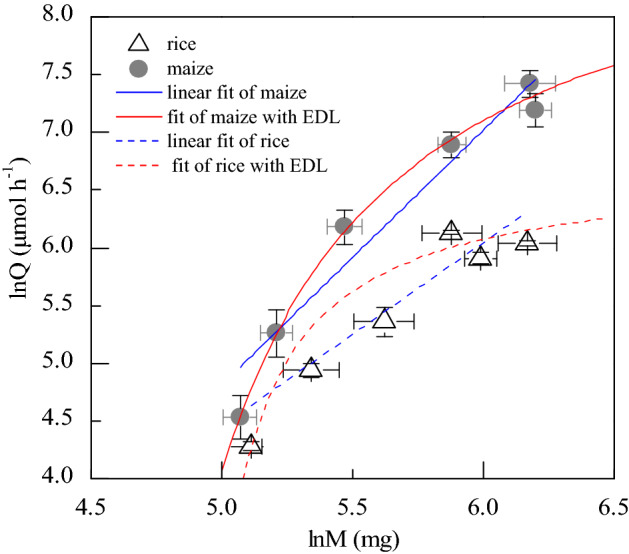
The non-loglinear relationship between ln*Q* and ln*M* at individual level in maize and rice. The total sample number of each treatment is fifteen.

The interdependent prediction (Eq. 4) was supported by the relationship between relative photosynthetic assimilation (ln*Q*) and growth (ln*M*) (Fig. 4). Meanwhile, the curvatures of the scaling between ln*Q* and ln*M* were different in rice and maize, because different species had different *K*_*q*_ and *K*_*m*_ (Eqs. 2, 3, Fig. 3). The effect of body size on relative rate of metabolism followed our assumptions at individual level (Fig. 4). The AIC values of traditional law were higher than that of the enzyme-driven law in rice and maize, respectively (Table 1). While the R^2^ value of enzyme driven law were also higher than that of traditional law in rice and maize, which matched and verified with AIC value. It meant that enzyme-driven law was better than traditional law.

**Table 1:** Comparisons of the performance between traditional power law and enzyme-driven law with Akaike's information criterion (AIC) in rice and maize.

Model	Rice		Maize	
	AIC	R^2^	AIC	R^2^
Traditional power law	41.56	0.70	20.23	0.92
Enzyme-driven law (Eq. 4)	37.54	0.84	12.66	0.98

## Discussion

The enzyme-driven law had been tested gradually from enzymatic (Eq. 1, Fig. 1), resource response (Eqs. 2, 3, Figs. 2, 3) to photosynthetic scaling (Eq. 4, Fig. 4). The AIC value indicated the enzyme-driven law (Eq. 4) was better than the traditional power law (although the traditional power equation could also be derived from the enzyme-driven hypothesis under special conditions (Eq. 5). The enzymatic dynamics had been used to describe the scaling of the metabolic balance in the micro algae of oceans^10,11^, in which the effect of body size on the metabolism had not been considered. However, the remarkable^36^ effects of body size on the metabolism^35^ could not be ignored. It is widely acceptable that both the metabolism and growth are a series of enzymatic process, which are constrained by the key enzymatic activities^37^, so it is reasonable to deduce the general law of metabolic scaling. The enzyme-driven law may be the origin of various metabolic scaling.

The AIC comparison shown that enzyme-driven law was better (Table 1) than the power law for the photosynthetic scaling. As these two equations could be deduced from enzyme-driven mechanism (Eq. 5), the enzyme-driven law might be more general than the traditional power law in photosynthetic scaling. Instead, previous studies on the curvilinear scaling were based on traditional power law with incorporation of other terms that derived from statistics^25–27^ or the energy dynamics^13,28^. The constraint factor of limited resource(s) have been considered in metabolic level boundary hypothesis, which was used to explain the variations of scaling exponent against metabolic level^30^. Moreover, the similar nonlinear pattern of metabolic scaling had also been found in teleost fish^38^. Meanwhile, a model of growth scaling was used to describe the growth of many diverse species proposed with parameter-less curve^39^. In addition, the polynomial equation of energy dynamics had been used to describe the nonlinear curve of active metabolic scaling during ontogeny in birds and mammals^28^. Instead, our equation (Eq. 4) could be used to describe the active and maintenance metabolic scaling, either linear or nonlinear pattern of scaling at log-scale. Overall, two kinds of linear and nonlinear pattern of photosynthetic scaling could be described by enzyme-driven law (Eqs. 4, 5), which might promote the quantitative integration between biochemical mechanism and ecological scaling.

## Material and methods

### Material and treatments

Maize (*Zea mays L. Zhefengnuo NO.3*) and rice (*Oryza sativa L. Nipponbare*) are representative species of the C_4_^/^C_3_ photosynthetic pathways^33,34^ and therefore were used in this study. Seedlings of two leaves were cultivated in black plastic buckets filled with Hoagland solution (pH 6.0) under a series of water potentials, which were adjusted to using polyethyleneglycol 6,000 (PEG-6000). Before the stage of two leaves, photosynthetic photon flux density (PPFD) and temperature were controlled in a stepwise diurnal sequence, with daily PPFD of 300 μmol m^−2^ s^−1^, and a photoperiod of 16 h. Temperature varied between 25 ℃/daily and 23 ℃/might.

### Methods of measurements

A portable open system (LI-6400; Li-Cor, Inc., Lincoln, NE, USA), equipped with a CO_2_ mixer, 30 mm × 20 mm chamber and red-blue LED light source (6400-2B) was used for the measurement of photosynthetic rate. Sample CO_2_, block temperature and photosynthetic active radiation (PAR) were set at 400 μmol mol^−1^, 36 ℃ and 1,200 μmol m^−2^ s^−1^ respectively. Dry weights of plants were obtained by oven drying at 105 °C for 30 min and then at 65 °C for 48 h.

The fluid enzymes were extracted by the modified li-ren’s method^42^. 0.2 g of leaf was put into a precooled mortar, then 100 mmol/ L of precooled Tris–HCl buffer (contained 7 mmol/L β-Mercaptoethanol, 1 mmol/ L EDTA, 5% glycerol and 1% PVP, pH 8.2); This sample was then centrifuged 4 ℃ for 20 min at 15,000 r/m. Then, we used supernatant fluid to measure the activity of RuBPCase and PEPCase with spectrophotometry^43^.

### Models

According to metabolism is a series of enzymatic processes^37^, we hypothesized that the relative change in photosynthetic assimilation (*dQ/Q*) was constrained by the change in key enzymatic activity (*dv*_*q*_) at individual level, including the effects of body size^8,40^. Hence, the relationship between photosynthetic assimilation rate (*Q*) and the key enzymatic activities (*v*) was obtained:

1 $$ \frac{dQ}{Q} \le dv_{q} $$

The relationship between *v* and substrate concentration *S* was assumed to abide by the Michaelis–Menten equation, therefore the relationship between *Q* and *S* can be described as follows:

2 $$ \ln Q \le \frac{{\ln Q_{m} \times S}}{{K_{q} + S}} $$

where ln*Q*_*m*_ is the maximum metabolic rate when *S* approaches saturation*, K*_*q*_ is a half-saturation constant (i.e., the substrate concentration at which the rate of substrate conversion is equal to ln*Q*_*m*_/2), R represent resource. *S* = *R* *−* *R*_0_, ln*Q*_0_ = ln*Q* when *S* equals zero.

We could hypothesize that the relative change in growth ($\frac{dm}{m}$) was also constrained by change in the key enzyme activity (*dv*_g_)^41^, which included the effects of body size on the growth^8,40^. The substrate-dependent equation of logarithmic body mass can be obtained by the integration of $\frac{dm}{m} \le dv_{g}$:

3 $$ \ln M \le \frac{{\ln M_{m} \times S}}{{K_{m} + S}} + \ln M_{0} $$

where ln*M*_*m*_ is the maximum body mass when *S* approaches saturation. ln*M* = ln*M*_0_ when *S* = 0, *K*_*m*_ is the half-saturation constant*.*

The enzyme-driven law of photosynthetic scaling were obtained by integrating Eqs. 2 and 3:

1. The interdependent relationship between *lnQ* and *lnM* was obtained When *K*_*q*_ ≠ *K*_*m*_:
  4 $$ \ln Q \le \frac{{\ln Q_{Mm} \times (\ln M - \ln M_{0} )}}{{K_{qm} + (\ln M - \ln M_{0} )}} + \ln Q_{0} $$
  where $\ln Q_{Mm} = \frac{{K_{m} \times \ln Q_{m} }}{{K_{m} - K_{q} }}$, $K_{qm} = \frac{{K_{q} \times \ln M_{m} }}{{K_{m} - K_{q} }}$, it displayed that ln*Q* varied a saturation curve with ln*M*
2. The log-transformed power equation was obtained, that is, *lnQ* increases linearly with *lnM* when *K*_*q*_ = *K*_*m*_:
  5 $$ \ln Q \le \frac{{\ln Q_{m} }}{{\ln M_{m} }}\ln M + \ln Q_{0} - \frac{{\ln Q_{m} }}{{\ln M_{m} }}\ln M_{0} $$
  where ln*Qm*/ln*Mm* is the exponent in the original power equation. Equation 5 shows that the famous traditional power law is a special form of the enzyme dynamics. Equations 4 and 5 both could be called as the enzyme-driven law of metabolic scaling.

### Statistical analysis

All the mathematical regressions and statistical analysis were performed in Origin 8.5. The methods were compared by the Akaike's information criterion (AIC)^39^. The model with the lowest AIC was regarded as the best representation of a curve^44^.
